# Unlocking the secrets of trace amine-associated receptor 1 agonists: new horizon in neuropsychiatric treatment

**DOI:** 10.3389/fpsyt.2024.1464550

**Published:** 2024-10-31

**Authors:** Britto Shajan, Tarun Bastiampillai, Shane D. Hellyer, Pramod C. Nair

**Affiliations:** ^1^ Discipline of Clinical Pharmacology, College of Medicine and Public Health, Flinders University, Adelaide, SA, Australia; ^2^ Department of Psychiatry, Monash University, Parkville, Melbourne, VIC, Australia; ^3^ Flinders Health and Medical Research Institute (FHMRI) College of Medicine and Public Health, Flinders University, Adelaide, SA, Australia; ^4^ Drug Discovery Biology, Monash Institute of Pharmaceutical Sciences, Monash University, Melbourne, VIC, Australia; ^5^ South Australian Health and Medical Research Institute (SAHMRI), University of Adelaide, Adelaide, SA, Australia; ^6^ Discipline of Medicine, Adelaide Medical School, University of Adelaide, Adelaide, SA, Australia

**Keywords:** GPCR (G protein-coupled receptor), TAAR1 (trace amine-associated receptor 1), drug discovery, drug repurposing, signalling bias, schizophrenia, depression and bipolar disorder

## Abstract

For over seven decades, dopamine receptor 2 (D_2_ receptor) antagonists remained the mainstay treatment for neuropsychiatric disorders. Although it is effective for treating hyperdopaminergic symptoms, it is often ineffective for treating negative and cognitive deficits. Trace amine-associated receptor 1 (TAAR1) is a novel, pharmacological target in the treatment of schizophrenia and other neuropsychiatric conditions. Several TAAR1 agonists are currently being developed and are in various stages of clinical and preclinical development. Previous efforts to identify TAAR1 agonists have been hampered by challenges in pharmacological characterisation, the absence of experimentally determined structures, and species-specific preferences in ligand binding and recognition. Further, poor insights into the functional selectivity of the receptor led to the characterisation of ligands with analogous signalling mechanisms. Such approaches limited the understanding of divergent receptor signalling and their potential clinical utility. Recent cryogenic electron microscopic (cryo-EM) structures of human and mouse TAAR1 (hTAAR1 and mTAAR1, respectively) in complex with agonists and G proteins have revealed detailed atomic insights into the binding pockets, binding interactions and binding modes of several agonists including endogenous trace amines (β-phenylethylamine, 3-Iodothyronamine), psychostimulants (amphetamine, methamphetamine), clinical compounds (ulotaront, ralmitaront) and repurposed drugs (fenoldopam). The *in vitro* screening of drug libraries has also led to the discovery of novel TAAR1 agonists (asenapine, guanabenz, guanfacine) which can be used in clinical trials or further developed to treat different neuropsychiatric conditions. Furthermore, an understanding of unappreciated signalling mechanisms (Gq, Gs/Gq) by TAAR1 agonists has come to light with the discovery of selective compounds to treat schizophrenia-like phenotypes. In this review, we discuss the emergence of structure-based approaches in the discovery of novel TAAR1 agonists through drug repurposing strategies and structure-guided designs. Additionally, we discuss the functional selectivity of TAAR1 signalling, which provides important clues for developing disorder-specific compounds.

## Introduction

Neuropsychiatric disorders such as schizophrenia are severe psychiatric conditions associated with a high personal and societal burden (1). For over seven decades, the pharmacological interventions have mainly involved antipsychotic drugs that act primarily by blocking the post-synaptic dopamine D_2_ receptors (2). These therapeutics have been somewhat successful in treating positive symptoms such as hallucinations, delusions, and more, but are ineffective in treating negative symptoms such as reduced motivation, interest, and expressive functions (3). Moreover, current antipsychotics have divergent side effect profiles ranging from sedation to life-threatening issues such as agranulocytosis (4). The common extrapyramidal symptoms (affecting movement and coordination) associated with first-generation antipsychotics have been improved with newer atypical antipsychotics, which show both D_2_ and 5-hydroxytryptamine type 2A (5HT_2A_) occupancy (5). However, adverse effects remain a major limitation, with nearly all patients experiencing some form of metabolic side effects such as weight gain and other comorbidities, contributing to a lack of treatment adherence (6, 7). Such limitations stimulated a growing interest in discovering and developing non-D_2_-based treatment strategies with superior clinical efficacy and fewer side effects (8–13).

Recent studies have implicated non-dopaminergic pathways in neuropsychiatric conditions (8–13). Trace amine-associated receptors (TAARs) represent a recently discovered superfamily of aminergic receptors, belonging to the class A family of G protein-coupled receptors (GPCRs). In humans, TAARs are encoded by the *Taar* gene mapped to human chromosome 6q23.2, encoding six functional genes (TAAR1, 2, 5, 6, 8 and 9), with isoform-dependent TAAR expression in central and peripheral tissues (14, 15). They are highly selective for trace amines, which are biogenic amines found in low concentrations (1-100 ng/g of tissue) (16–18). TAAR1 also exhibits an affinity for neurotransmitters, secondary metabolites, central nervous system stimulants (e.g. amphetamines) and other exogenous molecules. Since the discovery of TAARs, there has been a growing interest in TAAR1 as a potential neuropsychiatry target (19–21). Unlike other TAARs which are primarily expressed in olfactory tissues, TAAR1 shows no expression in the olfactory epithelium (22). TAAR1 instead displays significant expression in multiple brain regions, where it is highly expressed in monoaminergic neurons and modulates the activity of typical neurotransmitters including dopamine, glutamate and serotonin (20). Here TAAR1 function is critical as TAAR1 knockout (TAAR1-KO) mouse models broadly display typical schizophrenia-like symptoms including hyperdopaminergia, behavioural hypersensitivity, predisposition to substance addiction, impaired cognition and disrupted locomotive functioning (23–27). Several clinical and preclinical candidate molecules targeting TAAR1, along with genetic and pharmacological models mimicking neuropsychiatric conditions are being investigated to characterise TAAR1’s contribution to neuropsychiatric pathologies. For example, early work with synthetic TAAR1 agonists showed Ro5166017 has both anxiolytic and antipsychotic-like activity in mouse hyperdopaminergic and hypoglutamatergic models (28). These effects were only present in wild-type mice and blunted in TAAR1-KO models, indicating a primarily TAAR1-mediated effect and highlighting the therapeutic potential of targeting TAAR1. Such findings encouraged a rapid increase in interest in TAAR1 and the development of synthetic agonists for the treatment of neuropsychiatric disorders such as schizophrenia (20). In parallel, there has been increased interest in determining the utility of TAAR1 and its agonists in treating other conditions including Parkinson’s disease, depression, post-traumatic stress disorder and a few others (29–33). This, along with the development of new technologies allowing molecular-level detail of receptor-agonist interactions, has resulted in divergent strategies being employed to characterise new TAAR1 ligands (34, 35). In this mini-review, we discuss emerging strategies for designing, discovering, and developing novel TAAR1 agonists. In particular, we highlight the trend of using cryo-EM structures and delineating novel signalling mechanisms to develop TAAR1 agonists with optimised pharmacological profiles to treat schizophrenia-like phenotypes.

## Discovery of TAAR1 agonists using traditional approaches

Early pharmacological characterisation of TAAR1 and novel agonist discovery using *in vitro* models was hindered by several challenges. In recombinant cell systems, TAAR1 is primarily expressed intracellularly and also exhibits an inadequate expression level for pharmacological profiling (36, 37). In HEK293T cells, wild-type human TAAR1 is believed to lack asparagine-linked N-glycosylation that is critical for membrane expression and stability. A pioneering study by Barak and colleagues suggested that the lack of N-glycosylation causes the protein to degrade and the addition of the first nine amino acids of the human β2-adrenergic receptor remedies the lack of membrane expression. Furthermore, the species-specific activity among rat, mouse, and human TAAR1 orthologues despite sharing a high level of sequence similarity posed issues in structure-function studies. 3-Iodothyronamine (T1AM) is 10-fold more potent at rat TAAR1 (rTAAR1) than mTAAR1 and over 15-fold more potent than hTAAR1 (38, 39). These interspecies differences were later found to extend to several other endogenous and exogenous TAAR1 agonists (39, 40). Such insights have inspired a detailed study of molecular determinants underlying species-specific variance, highlighting several non-conserved residues contributing to the potency of TAAR1 agonists (41, 42).

**Figure 1:**
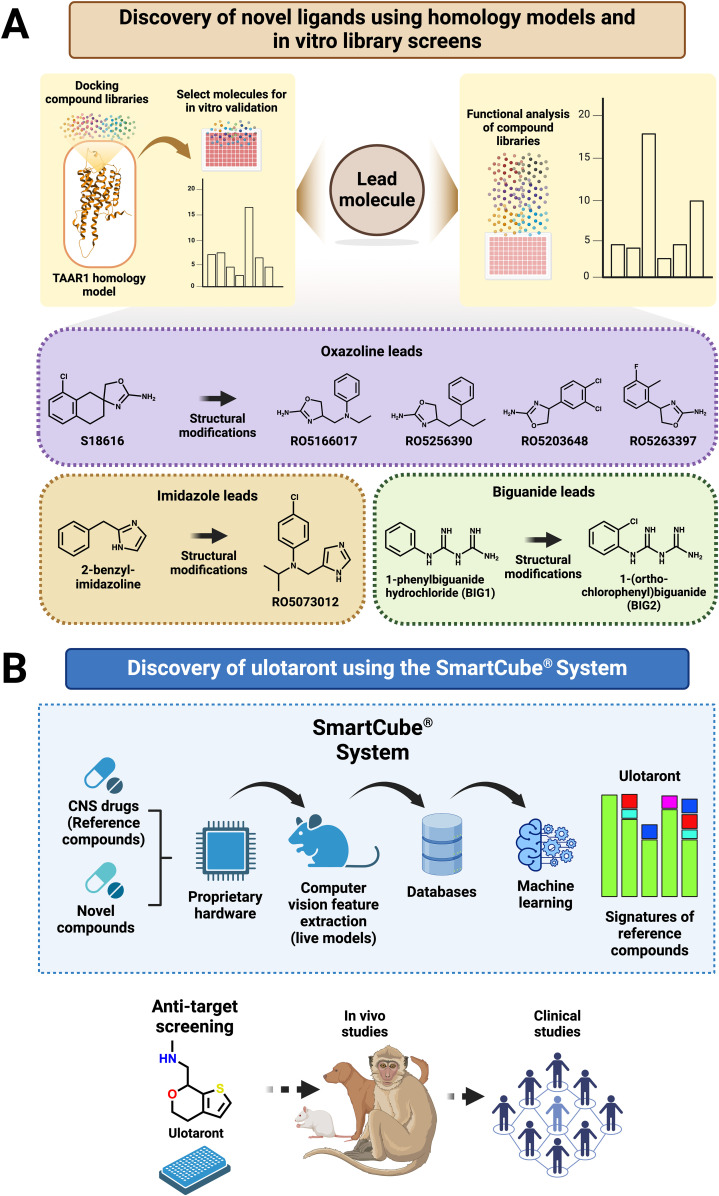
Discovery of TAAR1 agonist using TAAR1 homology model, *in vitro* screens and SmartCube® system. **(A)** Identification of different lead compounds using TAAR1 homology models and *in vitro* functional assays (e.g. oxazolines, imidazole and biguanides). **(B)** Utilisation of CNS drugs to identify new drug-like compounds with similar symptom-relief properties using the SmartCube® system. *In vitro* studies describing the non-D2-like mechanism of ulotaront, preclinical and clinical assessment of ulotaront for treating neuropsychiatric disorders.

The endogenous TAAR1 agonist scaffold influenced early developmental strategies for novel TAAR1 ligands. For example, early rational drug design used T1AM as a scaffold, resulting in the discovery of the first ‘superagonist’ and antagonist for rTAAR1 (43). The typical scaffold core features an aromatic ring linked to an amino group, an arrangement that is noticeable in several TAAR1 agonists based on oxazoline, imidazole and biguanide classes of compounds (**Figure 1A**) (44, 45). Several leads were identified and optimised using iterative modifications based on such compounds. Of note is S18616, an α_2_-adrenergic receptor agonist, that facilitated the discovery of Ro5166017 and other selective TAAR1 agonists (46–48). Additionally, chemicals with morpholine and carboxamide scaffolds were shown to display potent m/rTAAR1 activity (48). Furthermore, *in silico* methods and TAAR1 homology models provided key insights into TAAR1 structure-function (45, 49–51). Such techniques have expedited developmental timelines and facilitated rational drug design. Approaches such as molecular docking have enabled the virtual screening of large compound libraries and contributed to the discovery of new scaffolds, such as biguanide-based compounds whose agonist activities were validated with cyclic adenosine monophosphate (cAMP) functional assays (**Figure 1A**) (45). Traditionally, Gs-G protein signalling through cAMP was considered a key pathway for identifying TAAR1 agonists *in vitro*, and a lack of response in cAMP assays was interpreted as a lack of TAAR1 affinity. This rationalised the use of cAMP-sensitive Enzyme-Linked Immunosorbent Assay and biosensor-based assays as the standard to infer the stimulative strength of ligands *in vitro.* Whilst functional selectivity has been described for many class A GPCRs, the body of studies investigating the same for TAAR1 remained limited. Interestingly, Gq-based activities for TAAR1 were described previously (52). However, previous studies predominantly investigated ligands with Gs-based activity, thus limiting TAAR1 agonists with specific signalling pathways (53).

Using a combination of in silico pharmacology and cAMP-targeted validation techniques in previous years saw a steady increase in the characterisation of novel TAAR1 agonists. In this space, TAAR1 targeted compounds developed by Hoffmann-La Roche (referred to as “Roche or Ro”) and Sunovion Pharmaceuticals rapidly gained significance in neuropsychiatry due to their efficacy in preclinical and early clinical stages (28, 54). In most cases, lead compounds were identified by screening several in-house compound libraries using cAMP-based assays, followed by lead optimisation. Select compounds were tested in recombinant and native cell lines and their efficacy was later established in multiple animal species, including primates (55). One of the compounds discovered through such screening strategies was ralmitaront (Ro6889450), a partial agonist that was trialled in phase II clinical trials for treating schizophrenia (ClinicalTrials.gov ID: NCT03669640). However, ralmitaront failed such trials due to high placebo effects (56). Similarly, Ro5263397 entered phase I clinical trials but performed poorly due to a splicing polymorphism in a drug-metabolising enzyme among the trial population (57). Furthermore, several additional Roche compounds were tested, and some were demonstrated to be beneficial in treating psychosis, addiction, depression, cognition and sleep-related disorders in preclinical models, which have been detailed in previous reviews (20, 55, 58).

While previous approaches reflect the traditional “lead-hit optimisation” strategy, the discovery of select TAAR1 compounds was through a rather unique approach (51, 59). Most neuropsychiatric conditions are polygenic and pleiotropic, which brings significant challenges to traditional target-based strategies (60, 61). Instead, selecting a compound that elicits a desired therapeutic response (e.g. decreased hyperlocomotion), in contrast to developing and testing the activity at a pre-defined biological target (e.g. D_2_ receptor) seemed more appropriate. The SmartCube^®^ system, a high throughput mouse behavioural platform that harnesses various computer algorithms and machine learning tools to detect variations in behavioural response, was utilised in the discovery of the TAAR1 agonist ulotaront (SEP-363856) (**Figure 1B)** (62). Ulotaront demonstrated signatures associated with antipsychotic activity and modulating sleep patterns. Furthermore, off-target screening ensured that ulotaront did not have activity at D_2_ and 5HT_2A_ receptors, which are the targets of the current atypical antipsychotics (59). Given such robust preclinical success and novel targeting profile, ulotaront was granted a US FDA breakthrough therapy designation, noted as a potential paradigm-shifting compound in schizophrenia treatment and meeting endpoints of multiple phase I and II clinical trials (19, 63, 64). Ultimately, ulotaront failed to meet the primary endpoint in two phase III clinical trials for schizophrenia, again thought to be due to high placebo effects. However, ulotaront is currently being trialled (Phase II/III) for other neuropsychiatric conditions including major depression, generalised anxiety disorders and Parkinson’s disease psychosis (ClinicalTrials.gov IDs: NCT05593029, NCT05729373, NCT05015673, respectively).

## Discovery of novel TAAR1 agonists through binding pocket and pharmacophore-guided strategies

**Figure 2:**
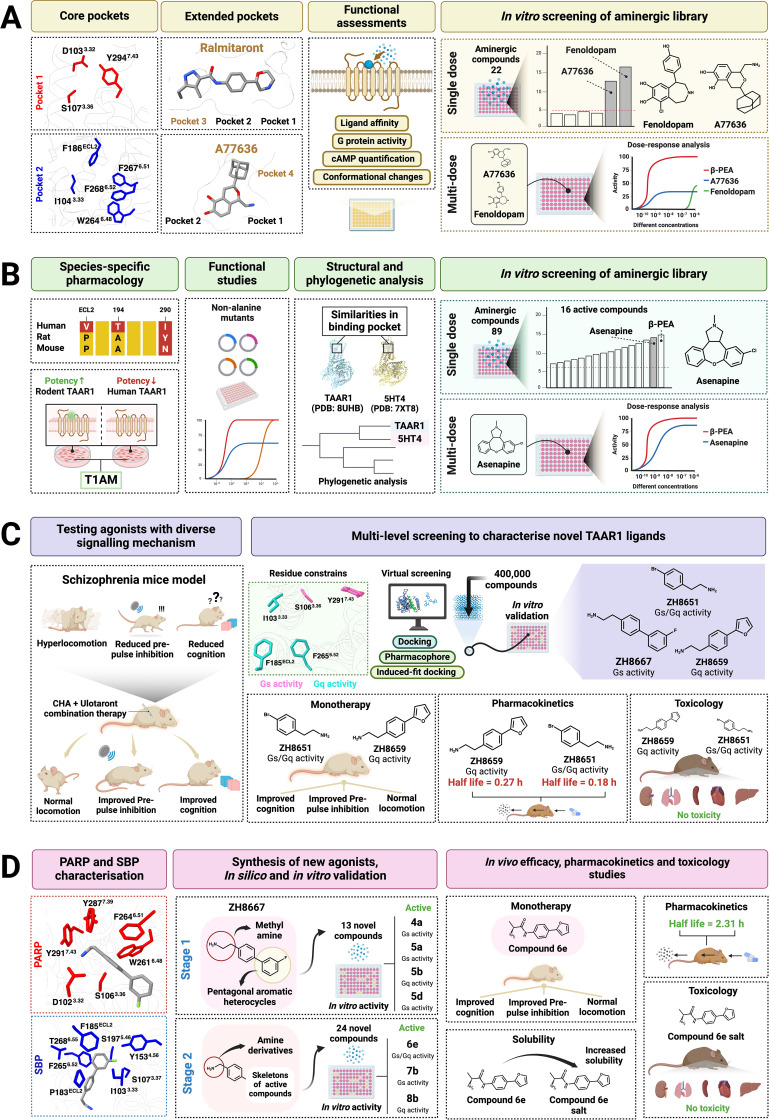
Discovery of novel TAAR1 agonists utilising cryo-EM structures. **(A)** Characterisation of core and extended binding modes of TAAR1, describing the role of critical binding site residues using divergent functional assays, and identification of fenoldopam and A77636 as TAAR1 agonists from an *in vitro* library screening of 22 compounds derived from a synthetic database (34). **(B)** Assessment of non-alanine binding site mutations, elucidation of TAAR1 structural homology with aminergic receptors and identification of asenapine as a potent TAAR1 agonist from an *in vitro* library screening of 89 aminergic compounds (35). **(C)** *In silico*, *in vitro* and *in vivo* models demonstrating the utility of TAAR1 ligands with atypical signalling properties. Identification of ZH8667, ZH8651, and ZH8659 from a large virtual screening of in-house libraries, and pharmacology characterisation in human cell lines and schizophrenia mice models (70). **(D)** Discovery of a compound 6e from ZH8667 using scaffold hopping, and pharmacological assessment of compound 6e in human cell lines and schizophrenia mice models (84).

Previous studies on TAAR1 structure and function relied on comparative models based on other GPCR structures (15, 50, 65–68). However, several recent studies have resolved multiple cryo-EM structures of both mTAAR1 and hTAAR1 in complex with G proteins and in the presence of various endogenous and synthetic agonists (34, 35, 69–71). TAAR1 structures are analogous to other GPCR-G protein complexes, featuring a canonical architecture that includes extracellular loop 2 (ECL2) adopting a ‘lid’ like conformation shielding the orthosteric site, a G protein heterotrimer in contact with cytoplasmic transmembrane (TM) helices, an elongated TM5 and a shorter TM6 (34). In general, the orthosteric site demonstrated high plasticity, accommodating ligands with divergent molecular scaffolds. The core binding pocket was formed by pockets 1 (D103^3.32^, S107^3.36^, Y294^7.43^, superscript numbering based on Ballesteros and Weinstein numbering scheme) and pocket 2 (I104^3.33^, F186^ECL2^, W264^6.48^, F267^6.51^ and F268^6.52^) residues (**Figure 2A**). In pocket 1, the D103^3.32^ establishes an ionic bond with positively charged amines and the larger pocket 2 facilitates recognition and stabilisation of the hydrophobic core of the ligands. In addition, two extended binding modes, involving pockets 3 and 4 (exemplified by ralmitaront and A77636, respectively), were characterised, implying plasticity of the TAAR1 binding site for accommodating scaffolds outside of the typical pharmacophore features represented by the previously known TAAR1 ligands (**Figure 2A**) (72). More recently, the binding modes of lysergic acid diethylamide (LSD) and Ro5263397 were described to concur with this observation. Jiang et al. described that the plasticity of the binding pocket and the constraint imposed by ECL2 creates a unique binding pocket that is distinct from other aminergic receptors (71). Therefore, recent molecular findings offer valuable insights for future drug discoveries and ultimately contribute to finding molecules with clinical applications.

The poly-pharmacological nature of clinical candidate ulotaront has also been further explored using cryo-EM by Xu et al. (34). In TAAR1 and 5-hydroxytryptamine type 1A structures, ulotaront binding modes appeared analogous, despite their sequence dissimilarities (34). Moreover, a large number of residues in the TAAR1 binding pocket are also non-conserved within the TAAR family, including residues in core pockets 1 and 2 (34). Considering this, it was reasoned that ligands possessing a typical structural core, similar to those expressed by trace amines, may exhibit activity at TAAR1. Broadly, this encompasses a phenyl-ethylamine or -methylamine backbone, critical for establishing interactions for ligand recognition and receptor activation (34, 35, 69–71). Furthermore, the non-conserved nature of binding site residues was hypothesised to allow divergent agonists to occupy the TAAR1 receptor binding site (34). As such, 22 compounds from a synthetic organic database based on dopaminergic, serotoninergic, and adrenergic receptors, including several antipsychotics and experimental compounds, were screened by Xu et al. for TAAR1 Gs activity (34). Here, cAMP assays identified fenoldopam (D1 dopamine receptor partial agonist and antihypertensive drug) and A77636 (an investigational compound) as agonists for TAAR1 Gs signalling (34). cAMP concentration-response analysis demonstrated A77636 to be 1000-fold more potent than fenoldopam. Furthermore, the maximal responses induced by both A77636 and fenoldopam were lower than both β-phenylethylamine (PEA) and ulotaront, suggesting partial agonism of TAAR1. Fenoldopam displayed a higher response relative to A77636 but not more than 50% in the concentrations tested. Along with fenoldopam, other groups have also identified antihypertensive drugs (guanabenz and guanfacine; α2 adrenergic receptor agonists) as TAAR1 agonists. This suggests that drug repurposing may be a viable approach for identifying novel TAAR1 agonists (73).

## TAAR1 structure and GPCR homology guided ligand characterisation

The TAAR1-Gs structure resolved by Zilberg et al. (35) closely resembles other reported structures (34, 35, 69–71). Except for D103^3.32^, F267^6.51^, F268^6.52^ and Y294^7.43^, all other residues at the TAAR1 binding site lack conservation compared to other aminergic GPCRs (35). Substitution of conserved and non-conserved residues resulted in broad effects on agonist-induced cAMP accumulation (35). The substitution of the key residue D103^3.32^ into asparagine (D103^3.32^N) indiscriminately abolished its response to all ligands and W264^6.48^F demonstrated a significant loss in agonist potency, highlighting them as key determinants in agonist binding at hTAAR1 (35). Further sequence comparison of hTAAR1 (e.g. V184^ECL2^, T194^5.42^, I290^7.39^) with rodent TAAR1 (e.g. P184^ECL2^, A194^5.42^, Y290^7.39^) highlighted dissimilarities at the orthosteric sites. Previous reports demonstrated that these residues influence the potency of several endogenous and synthetic compounds (74, 75). While most prior studies relied on alanine screens, here the effects of specific sequence variations were tested (**Figure 2B**). Substituting rodent TAAR1 residues for hTAAR1 radically influenced agonist potency. For instance, the substitution of I290^7.39^ for tyrosine (Y290^7.39^, from mTAAR1) increased potency for Ro5256390, Ro5263397 and ulotaront, while endogenous agonists such as PEA demonstrated a decrease in potency. Similarly, asparagine (N290^7.39^, rTAAR1) substitution resulted in increased potency for ulotaront and T1AM, without affecting the potency of PEA or tyramine. Meanwhile, substitution at T194^5.42^ to alanine (A194^5.42^, mTAAR1/rTAAR1), and V184^ECL2^ to proline (P184^ECL2^, mTAAR1/rTAAR1) influenced agonist potency in a ligand-dependent manner (35). These data highlight the large effect differences that a single residue can have on agonist activity between species, further highlighting the importance of TAAR1 cryo-EM structures and functional studies in TAAR1 drug discovery.

While TAAR1 has long been associated with aminergic receptors, the lack of experimental structures limited the ability to decipher its structural homology with others (76). An improved understanding of the structural homology and 3-dimensional characteristics, especially at the ligand binding site, offers valuable insights for *in silico* prediction of poly-pharmacology (77). Interestingly, phylogenetic analysis using sequence and structural data of class A17, A18 and A19 GPCRs demonstrated that human 5-hydroxytryptamine receptor 4 (5HT_4_), belonging to A19, shared the highest overall sequence similarity with TAAR1 (A17) (35). Structural alignment of TAAR1 and 5HT_4_ structures showed high structural homology in both G protein bound and unbound states. A comparison of binding pocket sequences of dopaminergic and serotoninergic families revealed significant similarities to TAAR1, even greater than those found with other TAARs (35). As catecholamines and exogenous aminergic ligands readily express poly-pharmacology, the newly discovered structural homology indicated that other aminergic ligands may also have off-target activities at TAAR1 (78). Subsequently, an evaluation of 89 aminergic drugs and chemicals (including several ergoline compounds, biogenic amines, beta-blockers, amphetamines, and antipsychotics) was conducted by Zilberg et al. to determine their ability to activate hTAAR1-Gs signalling via cAMP. A key discovery in this study was the identification of the atypical antipsychotic drug asenapine, which was found to potently activate TAAR1 to a similar level to PEA. While homology between TAAR and other aminergic ligands may present challenges with regard to ligand selectivity, it also provides opportunities to identify novel lead compounds that may be further optimised for TAAR1 activity.

A novel pathway to the role of TAAR1 in presynaptic dopamine modulation has been opened up by the discovery of current antipsychotics as potential TAAR1 activators (35). In the early literature, the notion of antipsychotics binding to TAAR1 coincided with the reduction in symptom relief observed in TAAR1-KO disease models (79). Whilst these certainly insinuated the “antipsychotics as TAAR1 ligands” paradigm, cAMP assays were unable to demonstrate any evidence of antipsychotic-induced cAMP accrual via TAAR1 (79). This, however, was reconsidered with the identification of functionally relevant heterodimers between TAAR1 and D_2_ receptors. Co-expression of D_2_/TAAR1 in a recombinant cell system enhanced PEA activity in the presence of D_2_ antagonist haloperidol, while TAAR1-KO animal models demonstrated a reduced response to haloperidol in a dose-dependent manner (80). In the absence of any other compelling evidence, the drug-induced symptom relief/lack of symptom relief was therefore implicated with dimerisation, rather than a direct activity at TAAR1. For years, this notion remained largely unquestioned. However recent evidence demonstrating direct activation of TAAR1 suggests that antipsychotics may also have TAAR1-mediated effects via alternative signalling pathways in addition to their traditional D_2_-blocking effects (80). Hence, future studies may benefit from including complementary assays to discover alternative signalling (such as calcium assays for Gq signalling), which may aid in discovering novel TAAR1 agonists. Moreover, understanding the intricate involvement of TAAR systems in the efficacy of drugs will potentiate future research focused on the clinical implications of TAAR1 mutations (81, 82).

## Discovery of novel TAAR1 agonists with different signalling mechanisms

Depending on the ligand, most GPCRs can conform to active conformations that facilitate their coupling with specific G proteins, activating G protein-dependant secondary messengers that produce specific cellular responses. Such variance in G protein selectivity is broadly defined as functional selectivity (83). Pharmacological profiling of divergent TAAR1 agonists using a G protein dissociation assay revealed new insights into TAAR1 functional selectivity. Contrary to previous understanding, several endogenous agonists of TAAR1 demonstrated activity through alternative signalling pathways that do not involve cAMP. For instance, while PEA and T1AM solely activated canonical Gs activation, cyclohexylamine (CHA) and isoamylamine only activated Gq signalling at both mTAAR1 and hTAAR1. Gq actives downstream signalling pathways mediated by phospholipase C (PLC), which hydrolyses phosphatidylinositol 4,5-bisphosphate (PIP2) to produce intracellular calcium mobilisation factor inositol 1,4,5-trisphosphate, and diacylglycerol, both secondary messengers of Ca^2+^ dependant protein kinase C (PKC). Meanwhile, trimethylamine (TMA) exhibited promiscuous signalling through all G proteins tested (Gs/q/i). A similar trend was observed with exogenous ligands, ulotaront, ralmitaront and *(S)*-amphetamine (AMPH). Ulotaront solely activated Gs, AMPH was coupled with functionally diametric coupling partners (Gs and Gi), and ralmitaront activated all G proteins similar to TMA (34). Further analysis of the G protein selectively revealed distinct receptor residues contributing to this selectivity. For instance, alanine mutation of S107^3.36^ and Y294^7.43^ abolished Gs activity, without imparting significant effects on Gi and Gq activity (34). Substitution of R83^2.64^, S183^ECL2^ and I290^7.39^ had a substantial effect on Gi but did not affect Gs signalling, and F186^ECL2^A and F267^6.51^A had more impact on Gq activity than Gs (34). Importantly, distinct G protein signalling is associated with the alleviation of symptoms in hypoglutamatergic models of schizophrenia, with preferential Gq-acting ligands such as CHA proving more efficacious, indicating the functional and therapeutic utility of these novel insights into TAAR1-G protein signalling (34, 70).

The delineation of TAAR1-Gq functionality was novel, as all the major TAAR1 agonists, whose actions are well-studied in multiple levels of animal models, have been developed based on their ability to activate TAAR1-Gs signalling. Given that both pathways seem to provide symptom relief, the utility of co-therapy using ulotaront and CHA was explored (**Figure 2C**) (70). A novel approach to administering TAAR1 agonists was to mimic a dual signalling activation mechanism, where both Gs and Gq signalling pathways are stimulated simultaneously. Ralmitaront, which failed phase II clinical trials and was previously believed to function via the Gs pathway alone, was recently shown to activate multiple Gi/s/q signalling pathways (34). However, concurrent coupling of functionally different signalling proteins (Gs and Gi) may be limiting, and dampen the effects mediated through cAMP signalling. In contrast, by administering ligands that target Gs and Gq independently, both signalling cascades can be transduced. In the mouse hypoglutamatergic model, co-therapy using CHA and ulotaront demonstrated symptom relief that was superior to ulotaront mono-treatment. As such, it was proposed that singular Gq activation and/or dual activation of Gq/Gs by TAAR1 may render superior clinical benefits to ulotaront alone. Further testing and reconsideration of previously discovered TAAR1 agonists for activation of alternative signalling pathways will likely provide new insights into TAAR1 ligand bias and signalling mechanisms that may be utilised in future design for novel agonists.

Indeed, leveraging the identification of preferential agonism, and newly resolved mTAAR1 structures, may facilitate the discovery of ligands with Gq or Gq/Gs signalling preferences. In the PEA-bound mTAAR1-Gs structure, an increased density in polar contacts with S106^3.36^ and hydrophobic contacts with Y291^7.43^ was noted compared to the CHA-bound mTAAR1-Gq structure (70). The increased bond density was proposed to rotate the hydroxymethyl group of S106^3.36^ and trigger a myriad of structural changes that ultimately facilitate the interaction between the cytosolic regions of mTAAR1 TM3 residues and C-terminus of Gs-heterotrimer. Hence it was reasoned that these residues may be essential for Gs selectivity in mTAAR1 (**Figure 2C**). In addition, stimulation of TAAR1 mutants (I103^3.33^A, F185^ECL2^A, or F265^6.52^A) using TMA, showed decreased activity for Gs and Gq coupling but not for Gi. Therefore, it was reasoned that I103^3.33^, F185^ECL2^ and F265^6.52^ may be critical for Gs/Gq selectivity in mTAAR1. Thus, after constraining S106^3.36^ and Y291^7.43^ for Gs activity, and I103^3.33^, F185^ECL2^ and F265^6.52^ for dual activation mechanisms, multilevel virtual screening was conducted on a library of 400,000 compounds. Compounds were initially docked using an induced fit docking model onto the mTAAR1 structure and further refined using binding energy calculation based on molecular mechanics generalised-born surface area (MM/GBSA) and ligand-receptor contacts. 54 compounds identified from docking studies were functionally tested using signalling assays for both Gs and Gq activity. This approach identified ligands with Gs-coupled (ZH8667) and Gq-coupled (ZH8659) activities, in addition to the dual agonist ZH8651, all of which were subsequently tested in mice models and replicated the effects of ulotaront and CHA as either mono or co-therapies (70).

While both ZH8651 and ZH8659 appeared to be strong candidates, both were limited by their pharmacokinetics, as they exhibited very short half-lives. However, given the promising endpoints from their primary study, more novel agonists designed based on ZH8667 were recently synthesised (**Figure 2D**) (84). Structurally, ZH8667 comprises a fluorobenzene linking to benzene, with an ethylamine chain. In mTAAR1 complexed with ZH8667, it was noted that this architecture enables extensive interactions with primary amine recognition pocket (PARP, D102^3.32^, S106^3.36^, W261^6.48^, F264^6.51^, Y287^7.39^ and Y291^7.43^) and second binding pocket (SBP, I103^3.33^, S107^3.37^, Y153^4.56^, P183^ECL2^, F185^ECL2^, S197^5.46^, F265^6.52^ and Y268^6.55^) residues (70, 84). The additional contacts in SBP were postulated to give ZH8667 its Gs activation properties. As such, developmental strategies were focused on synthesising a novel compound with minimal to no SBP interaction while maintaining all PARP interactions. Initial efforts aimed to reduce the physical size of the molecule and several iterative structural modifications produced four compounds with TAAR1-Gs activity (84). However, none demonstrated dual Gs/Gq activity, resulting in further modifications and resulting in agonists with single and dual G protein activity. Here, compounds 7b, 8b and 6e were identified as the most potent in their category (Gs, Gq and dual Gs/Gq, respectively). In MD simulations, 6e achieved superior binding and conformational stability at the mTAAR1-Gs complex compared to others. Subsequently, testing of these compounds in the hypoglutamatergic model of schizophrenia revealed compound 6e as superior in reducing hyperlocomotion and improving cognitive function, without inducing catalepsy. In pharmacokinetic evaluations, compound 6e demonstrated an improved terminal half-life compared to the parent compound. Moreover, no toxicity markers were found after chronic oral administration in healthy mice. Overall, this study demonstrates the structure-based rational design of ligands with preferential activation of select G proteins as an exciting new avenue for developing more efficacious TAAR1 agonists.

## Conclusion and future perspectives

TAAR1 agonists offer a new avenue for the treatment of neuropsychiatric conditions. The recent emergence of human and mouse TAAR1 structures in the presence of diverse agonists ranging from endogenous compounds to clinical candidates provides detailed atomic insights into TAAR1 binding site plasticity, ligand binding interactions, and binding modes. Such advancements pave the way to develop more selective drug-like compounds. Particularly, the identified structures provide significant insights into the structure-function of TAAR1 in the context of poly-pharmacology and species-specific differences in ligand recognition. Drug re-purposing strategies have also led to the identification of different existing drugs (asenapine, fenoldopam, guanfacine, guanabenz) as TAAR1 agonists which can serve as lead compounds for future drug discovery efforts for different neuropsychiatric disorders. Ligand bias in TAAR1 opens up new opportunities to explore novel signalling pathways to discover agonists that can activate different signalling pathways that have shown promise for treating mouse models with schizophrenia-like symptoms. Future studies on TAAR1 exploring unique signalling pathways may allow the discovery of novel compounds to treat different neuropsychiatric conditions. Based on these newly developed structural and biochemical observations of TAAR1, rational discovery of new ligands using computational and pharmacological approaches would be valuable in developing novel TAAR1 therapeutics.
